# Perioperative Individualized Goal Directed Therapy for Cardiac Surgery: A Historical-Prospective, Comparative Effectiveness Study

**DOI:** 10.3390/jcm10030400

**Published:** 2021-01-21

**Authors:** Davinder Ramsingh, Huayong Hu, Manshu Yan, Ryan Lauer, David Rabkin, Jason Gatling, Rosario Floridia, Mckinzey Martinez, Ihab Dorotta, Anees Razzouk

**Affiliations:** 1 Department of Anesthesiology, Loma Linda University Medical Center, Loma Linda, CA 92354, USA; dramsingh@llu.edu (D.R.); HHu@llu.edu (H.H.); rlauer@llu.edu (R.L.); jgatling@llu.edu (J.G.); MKMartinez@llu.edu (M.M.); idorotta@llu.edu (I.D.); 2 Department of Cardiothoracic Surgery, Loma Linda University Medical Center, Loma Linda, CA 92354, USA; DRabkin@llu.edu (D.R.); RFloridi@llu.edu (R.F.); arazzouk@llu.edu (A.R.)

**Keywords:** cardiac anesthesia, adult cardiac care, adult critical care, hemodynamic monitoring, goal directed therapy

## Abstract

Introduction: Cardiac surgery patients are at increased risk for post-operative complications and prolonged length of stay. Perioperative goal directed therapy (GDT) has demonstrated utility for non-cardiac surgery, however, GDT is not common for cardiac surgery. We initiated a quality improvement (QI) project focusing on the implementation of a GDT protocol, which was applied from the immediate post-bypass period into the intensive care unit (ICU). Our hypothesis was that this novel GDT protocol would decrease ICU length of stay and possibly improve postoperative outcomes. Methods: This was a historical prospective, QI study for patients undergoing cardiac surgery requiring cardiopulmonary bypass (CPB). Integral to the QI project was education towards all associated providers on the concepts related to GDT. The protocol involved identifying patient specific targets for cardiac index and mean arterial pressure. These targets were maintained from the post-CPB period to the first 12 h in the ICU. Statistical comparisons were performed between the year after GDT therapy was launched to the last two years prior to protocol implementation. The primary outcome was ICU length of stay. Results: There was a significant decrease in ICU length of stay when comparing the year after the protocol initiation to years prior, from a median of 6.19 days to 4 days (2017 vs. 2019, *p* < 0.0001), and a median of 5.88 days to 4 days (2018 vs. 2019, *p* < 0.0001). Secondary outcomes demonstrated a significant reduction in total administered volumes of inotropic medication(milrinone). All other vasopressors demonstrated no differences across years. Hospital length of stay comparisons did not demonstrate a significant reduction. Conclusion: These results suggest that an individualized goal directed therapy for cardiac surgery patients can reduce ICU length of stay and decrease amount of inotropic therapy.

## 1. Introduction

Cardiac surgery requiring cardiopulmonary bypass is a high-risk procedure. In recent years more patients with multiple comorbidities are undergoing cardiac surgery [1,2]. These high-risk patients often have limited physiologic reserve and are at increased risk for postoperative morbidity. Indeed, post-operative complication rates for cardiac surgery are high. Reported rates for major postoperative complications range from 15% to 30%, which include: stroke, renal failure, prolonged intubation, and sternal wound infection [3,4]. Additionally, approximately 19–45% of the cases may go through prolonged intensive care after open heart operation [5]. These indices suggest the continued need to evaluate areas of improvement.

The concept of goal-directed therapy (GDT), as it applies to hemodynamic (HD) optimization, is the titration of fluids, inotropes, and possible blood transfusions to achieve optimal oxygen delivery to the tissues. Often these protocols utilize flow-guided technologies to allow for more complete hemodynamic assessment in addition to routine blood pressure monitoring. For non-cardiac surgery, there are many randomized controlled trials demonstrating the utility of GDT to reduce length of stay (LOS) and postoperative complications across a variety of surgical patient populations [6], which has been further supported by several meta-analyses [7,8,9]. Indeed, this cumulative evidence has led to the incorporation of GDT principles into several clinical practice guidelines [10,11,12].

For cardiac surgery requiring cardiopulmonary bypass (CPB), the widespread application of GDT remains underdeveloped. This is despite research demonstrating the utility for intraoperative GDT over 25 years ago [13]. Over the years, several studies have demonstrated the utility of both intraoperative GDT for cardiac surgery [13,14] as well as postoperative GDT [15,16,17]. Additionally, hybrid GDT, which implemented strategies both intraoperatively and postoperatively, have also demonstrated an improvement in patient care [18,19]. Moreover, integration of these studies under several meta-analyses have demonstrated an improvement in GDT over standard treatment for overall complications and hospital length of stay [19,20]. These studies have supported the inclusion of GDT into recent Enhanced Recovery After Surgery Society Guidelines for Perioperative Care in Cardiac Surgery as a Class I, Level B–R recommendation [21].

As the evidence and recommendations continue to support the application of GDT for cardiac surgery, more emphasis should fall on the strategies to implement GDT care for cardiac surgery as a standard of care. Moreover, this strategy should integrate GDT protocolization across the intraoperative and postoperative period in the intensive care unit (ICU). Finally, recognition for the greater variability in target hemodynamic parameters for the cardiac surgery patient is another important point of consideration for designing cardiac surgery GDT protocols. Indeed, the current evidence has not thoroughly evaluated a hybrid GDT protocol utilizing individualized targets of flow and pressure.

In an effort to establish a GDT protocol as a standard of care for both the intraoperative and the ICU settings, we launched a multidisciplinary quality improvement initiative (QI) for patients undergoing cardiac surgery requiring cardiopulmonary bypass (CPB). This program was established under a principle that multidisciplinary education and communication pathways must be an integral component of GDT implementation. Additionally, we followed a concept that patient specific targets are also essential for impactful protocolization to provide hemodynamic optimization. The overall goal of this QI initiative was to evaluate the effectiveness of the systematic implementation of a peri-operative individualized GDT protocol on the postoperative length of stay in the ICU (ICU LOS) and on the incidence of postoperative complications on cardiac surgical patients.

## 2. Methods

The study was approved by the Institutional Review Board at Loma Linda University (IRB #5160253) prior to study launch as a propsective evaluation of QI project. The analytic plan was published on clinicaltrials.gov before data analysis (# NCT04458701). Patient consent was waived as this was a QI initiative evaluating the implementation of a GDT protocol for eligible patients as a new institutional standard of care. Since the study was initiated as a quality improvement (QI) project, it is reported following the Standards for Quality Improvement Reporting Excellence (SQUIRE guidelines) [22,23] and is presented as a historical prospective comparative effectiveness format following the GRACE (Good Research for Comparative Effectiveness) initiative principles and checklist [24,25]. The study was performed at a tertiary care teaching hospital.

All patients admitted from dates 1 February 2019 up to 31 December 2019 qualified for study inclusion if the following were meet: (1) the procedure was a first-start case, (2) the procedure was scheduled, (3) the procedure was considered non-emergent, (4) the operation required CPB, and (5) patients were expected to require an inotropic and/or vasoconstrictive agent for more than 12 h post-CPB. Patient demographics and baseline data was collected retrospectively from the electronic health records (EHR) for procedures conducted in 2017 and 2018. Patients less than 18 years of age, off-pump procedures, non-first-start or emergent classification, as well those who did not receive at least 12 h of inotropic and/or vasoconstrictive agents were excluded from the study.

### 2.1. Development of the Quality Improvement Initiative

**Figure 1:**
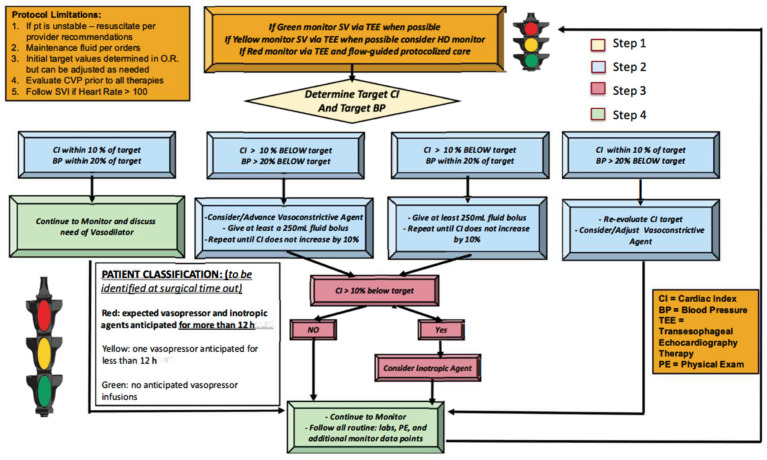
Cardiac hemodynamic optimization protocol. Legend: Stop light system utilized at surgical time out to identify patients with strong likelihood for requiring agents for more than 12 h (left lower box). Target CI and BP determined using multiple monitoring data within 45 min after separating from CPB, steps are followed depending on patient’s CI and BP range (flow chart algorithm; fluid challenge composed of 5% albumin or crystalloid, vasoconstrictive agent composed of norepinephrine, vasopressin, phenylephrine and epinephrine. Inotrope composed of milrinone).

Initial discussions occurred in early 2018 between cardiothoracic (CT) anesthesiology, critical care, and CT surgery for the development of a protocolized hemodynamic management strategy. Once an agreement was reached on the merits of the partnership, a multidisciplinary GDT planning team was assembled, consisting of: (1) three CT surgeons, (2) three CT anesthesiologists, (3) three CT intensivists, (4) two nurse practitioners, (5) two research assistants, (6) a case manager, (7) two patient safety officers, and (8) an information technology expert. Across multiple meetings this team developed a combined peri-operative hemodynamic optimization protocol (Figure 1).

A description of this protocol process is as follows: All non-emergent, first-start, cardiac surgery requiring CPB were evaluated. Surgery categories included CABG, aortic valve replacement, aortic aneurysm repair and adult congenital cardiac procedures (Table 1). All patients received general anesthesia with isoflurane and fentanyl maintenance. Pre-induction arterial lines were placed on all subjects according to institutional protocol for cardiac surgery, hemodynamic monitoring device (EV 1000 Edwards Lifesciences, Irvine, CA, USA) was connected upon arterial line placement recording parameters including cardiac index (CI), stroke volume index (SVI), systemic vascular resistance index (SVRI), stroke volume variation (SVV). Central lines were placed on all subjects for central venous pressure (CVP) monitoring and volume/medication infusion. Pulmonary artery catheter (PA catheter) was not universally placed but based on the patient’s underlying condition such as pulmonary hypertension, right heart failure and surgeon’s preference. Sedline brain function monitoring and near infra-red spectrometer (NIRS) were placed on all patients to monitor anesthetic depth as well as regional brain perfusion. Transesophageal echocardiograms were utilized on all subject for the intra operative period. At the time of the surgical time-out the CT anesthesia and surgery teams would evaluate if the patient was likely to qualify for the protocol using a “stop light”, green, yellow, red designation. Green signified a patient who is unlikely to require a vasopressor or inotropic agents for >12 h, yellow signified a possible likelihood for agents, and red signified a strong likelihood for requiring agents. Patients had their status reconsidered after separation from CPB, which would decide their inclusion in the QI project or not. A research assistant would monitor all first start cardiac cases for protocol inclusion. Once the patient was determined to be “red”, the intraoperative team would identify the patient’s target MAP and CI (Figure 1). The target CI and MAP were determined by anesthesia and surgical team using many factors, including patient’s comorbidities, pre-bypass HD monitoring data, post-bypass HD monitoring data, intra-operative event such as intra-operative hypotension, transesophageal echocardiography data, cerebral oximetry monitoring data, urine output, CPB time and surgical variables etc. Target CI and MAP were established within the first 45 min after separating from CPB when the intraoperative team identified HD stability. These targets were written on a HD datasheet that was attached to the HD monitor and included the patient’s target values along with space for hourly documentation of metric review for 12 h.

After the protocol was established, a three-month trial period was launched (November 2018 to January 2019). During this period providers involved in the patients intraoperative and ICU care received training on the protocol and review of the HD monitor (EV1000, Software Version:1.9, Edwards Lifesciences, Irvine, CA, USA). This included: (1) CT anesthesia, (2) CT surgery, (3) anesthesiology residents, (4) CT ICU nurses (day and night shifts), and (5) CT intensivists. Importantly, all HD equipment used for this QI project had been available at the study hospital for over 5 years prior to project initiation. However, a multidisciplinary training program for all the providers listed above had not been implemented prior to the QI launch.

### 2.2. Process Measures

To evaluate the impact of the QI initiative on patient care, we compared the pre-implementation period for two years (January 2018 through December 2018 and January 2017 through December 2017) to the post implementation period (February 2019 to December 2019). To ensure equality between the representative datasets, all data was retrieved from the EMR using the same screening criteria (adult patients, first-start case of the day, scheduled surgery, requiring cardiopulmonary bypass, patients expected to require an inotropic and/or vasoconstrictive agent for more than 12 h post-CPB). All outcome measures were retrieved through the same processes via review of electronic medical record (Epic Systems Corporation, Verona, WI, Italy).

### 2.3. Outcome Measures

The primary outcome measure was ICU length of stay (LOS), defined as the number of nights spent in the ICU. Criteria for ICU transfer included: (1) stability of vital signs with removal of invasive monitoring device (arterial catheter and central line removed if patient’s hemodynamic data within goal for more than 4 h without pressor/inotropic support) (2) removal of mechanical ventilation support for the past 12 h, (3) control of postoperative pain, and/or (4) absence of worsening organ function. The decision for ICU transfer was a collaborative one between the intensivist and CT surgeon. The secondary outcome measures included the major postoperative complications identified as: (1) stroke, (2) acute kidney injury (AKI) or acute renal failure (ARF, defined as a threefold or greater rise in creatinine or new dialysis requirement), (3) prolonged intubation (>24 h), and/or (4) sternal wound infection. Additional, secondary outcome measures included total length of hospital stay (in days) and 30-day hospital readmission. The decision for hospital discharge was made by the CT surgeon. All outcome assessors were blinded to group allocations among three groups. Importantly, protocol compliance was monitored by reviewing the completion of the HD datasheets from the ICU.

### 2.4. Analysis

During the QI period, edited HD datasheets were retrieved from the ICU for 92% of cases. Primary and secondary outcomes showing significant departures from normality were summarized using medians and inter-quartile ranges, otherwise means and standard deviations were used. The Hodges-Lehmann estimator (difference in median) and respective confidence intervals are presented in Table 1. This non-parametric estimator is used to give a shift estimate of the difference between the values in two independent sets of data. A two-sample t-test was used to evaluate all continuous numeric outcomes meeting parametric assumptions. A Mann-Whitney test was used for any numeric outcomes not meeting parametric assumptions. A Chi-Squared test was used to evaluate all categorical outcomes and categorical outcomes not meeting assumptions, were evaluated with a Fisher’s exact test. All primary and secondary outcomes were compared across study years (2017–2019, 2018–2019, and combined 2017/2018–2019) using multiple univariate tests without multiple testing correction. The primary outcome for this study was ICU length of stay (in days). The secondary outcomes include the following: (1) hospital length of stay (in days), (2) incidence of stroke, (3) incidence of AKI, (4) respiratory failure, (5) a surgical site infection, (6) composite complication score, (7) amount of total vasopressor medications (for milrinone, norepinephrine, phenylephrine, epinephrine, and vasopressin), (8) amount of total fluid and blood products administration and (9) 30-day hospital readmission. The incidence of clinical complications was based on the diagnosis documentation by ICU team per medical chart review. The Shapiro Wilk test was used to test for normally and the Levene’s test was used to check variance assumptions. All statistical analysis and graphics were conducted in R version 3.5.0 (Boston, MA, USA).

## 3. Results

Patient baseline demographics including, age, sex, weight, height, disposition metrics, comorbidities and surgical categories are reported in Table 1.

Comparison of the primary outcome marker, ICU LOS, showed a statistically significant reduction between the year in which the QI study was launched (2019) to both years prior, *p* value < 0.001. (Table 2) Secondary outcome comparisons of the total vasopressors and inotropic agents demonstrated insufficient evidence for a difference in total volume administration of: norepinephrine, phenylephrine, epinephrine, and vasopressin, across 2017, 2018, and 2019 groups, *p* value > 0.2). (Table 3). However, there was sufficient evidence indicating a reduction in milrinone total volume administered between 2019 and both 2018 and 2017, *p* < 0.01. (Table 3). Comparison of intraoperative blood product administration demonstrated a significant reduction in the units of fresh frozen plasma and platelets transfused between the 2017 to 2019 groups, *p* < 0.05. (Table 3) Comparison of IV fluid administration demonstrated a significant reduction in intraoperative IV fluid administration between both 2017 and 2018 data to 2019, *p* < 0.001.(Table 3) Additionally, comparison of ICU fluid administration demonstrated a significant increase in IV fluid administration between both 2017 and 2018 data to 2019, *p* < 0.001. (Table 3) Comparison of total IV fluid administration demonstrated a significant reduction when comparing 2017 to 2019 data only, *p* = 0.011. (Table 3) The rates of strokes, respiratory failure, AKI, surgical site infection, and a composite of these complications did not prove to be statistically significant across all comparisons. (Table 2). Lastly, this study found insufficient evidence to show a difference in hospital LOS and 30-day readmission across 2017 to 2019 as well as for 2018 to 2019.

## 4. Discussion

Under the conditions of this QI project, we present the positive impact on the postoperative course of cardiac surgical patients with the implementation of a hemodynamic management protocol. This protocol applied individualized targets from the intraoperative post-bypass period to the first twelve hours of the ICU admission and was developed in a multidisciplinary effort involving CT anesthesiology, CT surgery, and CT intensivists. Our findings support the works of others who have demonstrated the utility of goal directed therapy for cardiac surgery patients in both the operating room and ICU. Novel components of this project include the integration of a single protocol for both clinical settings that utilized individualized targets, along with a strong emphasis on education and training on HD technology and GDT concepts. Moreover, the project was intentionally implemented as a QI initiative to evaluate the ability to improve the standard of care at our academic center.

Although multiple hemodynamic optimization research studies have been undertaken in cardiac surgery, adoption of enhanced recovery principles has not grown at the same rate as non-cardiac procedures. This may be secondary to barriers unique to cardiac surgery such as lack of uniformity amongst procedures, emphasis on the value of individual practice preferences, and greater variability in comorbidities and hemodynamic status amongst patients compared to non-cardiac surgery. Our study demonstrates an approach to gain wide-spread adoption of an individualized GDT protocol for cardiac surgery patients at a tertiary care academic center. Over the 9-month period, prior to the QI protocol launch, we developed a training program that was utilized by all care team members. This program provided education on the principles of GDT, explanation of the established protocol, as well as instruction on HD monitors utilized for project. Indeed, despite the technologies being available for a significant period prior to the project launch, the QI team discovered a wide variability in experience/training on the HD monitors used for the study as well as with the physiologic concepts around GDT therapy during the three-month training period. However, with the implementation of a structured educational program, we were able to demonstrate a high rate of compliance as measured by review of the care-teams HD datasheets.

Our study differs from previous cardiac-surgery GDT projects in some key aspects. First, we report a novel “stoplight” system to identify and communicate patient’s risk classification. Second, the GDT protocol utilized for this study did not set universal absolute HD targets of flow and pressure. Rather, individualized targets of MAP and CI were utilized. These thresholds were determined intraoperatively, post-CPB, by the intraoperative team (surgery and anesthesiology) utilizing a variety of inputs including: patient’s comorbidity, pre-CPB and post-CPB HD data, echocardiography, cerebral oximetry, urine output, CPB duration and surgical variables. The concept of individualized hemodynamic management using patient’s baseline cardiac index in high-risk abdominal surgical patients had demonstrated to reduce postoperative complications [26]. And It is the authors’ belief, that identifying individualized targets was a key component of impacting patient improvement and supported the high compliance of GDT protocol adoption.

There has been study showing positive intraoperative fluid balance during cardiac surgery to be associated with decreased postoperative complications like AKI, however we believe that GDT in cardiac surgery is a complex one and fluid balance is only one part of the consideration [27,28]. Of note, our intervention 2019 GDT group demonstrated a significant alteration in IV fluid administration, both in intraoperatively and the ICU compared to historic data. Specifically, the administration of IV fluids was significantly reduced intraoperatively and significantly increased in the ICU. This significant alteration to historic IV fluid administration data demonstrates the impact of the GDT protocol on care practices. Additionally, it offers a possible mechanism for the reduction in ICU LOS by demonstrating the importance of the timing of fluid administration during the patient’s hospitalization. This concept has been well demonstrated in non-cardiac [29,30]. Moreover, our intervention GDT group demonstrated a reduction in milrinone usage compared to the years prior, which is in contrast to other studies that demonstrated an increase in inotropic agents in the intervention group. This is likely to the previous institutional preference to support a theoretical reduction of cardiac function post-surgery and demonstrates the utility of GDT protocols to avoid unnecessary medication delivery. Moreover, our study did not find a significant reduction in major complications of: AKI, stroke, respiratory failure, and surgical site infection. Importantly, our study was not powered to detect for an improvement in these metrics and others have shown an improvement in ICU LOS without showing a reduction in major operative complications [2].

This study has several limitations. As with all quality improvement studies, in comparison to randomized trials, our study aims to determine statistically significant associations between the intervention and the outcome, but cannot determine causations. Importantly, during the years of the study period there was no additional QI initiatives implemented for cardiac surgical patients. Moreover, all data was retrieved retrospectively with the same data collection methodology. Detection of complications were dependent on documentation by the health care providers in the patient care clinical documents. Nevertheless, potential differences in patient characteristics and risk scores between years is a notable limitation for this type of study design. The study sample size was determined by study inclusion criteria over the study period. Thus, no prospective power analysis was performed. The study may also have limited external validity given its single center design.

## 5. Conclusions

This study highlights the development of a perioperative individualized GDT protocol for cardiac surgery patients who are likely to require more than 12 h of pressor/inotropic support. The protocol was launched as a new institutional standard of care, which focused on education and training of all team members to ensure adoption and protocol compliance. Comparison of one year of the QI initiative to years prior demonstrated an improvement in ICU length of stay and decrease in inotropic therapy use.

## Figures and Tables

**Table 1:** Baseline characteristics and demographics of patients.

		Group 1 (2017) *n* = 175	Group 2 (2018) *n* = 217	Combined Group 1–2 (2017–2018) *n* = 392	Group 3 (2019) *n* = 158
Age (in years)	Median (IQR)	65.50 (15.25)	64.00 (14.00)	65.00 (15.00)	62.00 (15.75)
Sex Male:Female	Count (Percentage)	117 (67%):58 (33%)	135 (62%):82 (38%)	252 (64%):140 (36%)	113 (71.5%):45 (28.5%)
Weight (in kilograms)	Median (IQR)	80.10 (24.011)	82.79 (25.57)	81.65 (24.81)	84.45 (28.65)
Height (in centimeters)	Median (IQR)	170.18 (15.24)	167.64 (17.78)	170.18 (24.81)	172.70 (15.00)
Diabetes Mellitus (Type II)	Count (Percentage)	65 (37.1%)	81 (37.3)	146 (37.2)	75 (47.5)
Chronic Kidney Disease/ESRD	Count (Percentage)	26 (14.9%)/10 (5.7%)	48 (22.1%)/19 (8.8%)	74 (18.9%)/32 (7.4%)	39 (24.7%)/13 (8.2%)
Chronic Systolic Hear Failure (EF < 40%)	Count (Percentage)	20 (11.4%)	43 (19.8%)	63 (16.1%)	24 (15.2%)
Surgical Categories	Count (Percentage)				
CABG		103 (58%)	138 (64%)	241 (61.1%)	109 (69.2%)
AVR		39 (22%)	47 (22%)	86 (22%)	40 (25%)
Aortic Aneurysm Repair		7 (3.5%)	3 (1.5%)	10 (2%)	7 (4.2%)
ASD repair		1 (0.5%)	1 (0.5%)	2 (0.4%)	0 (0%)
Mitral valve repair		14 (8%)	23 (10.5%)	37 (9%)	0 (0%)
Other		11 (5.5%)	5 (2.5%)	16 (3.2%)	2 (1.2%)

IQR = interquartile range; ESRD= end stage renal disease; CABG = coronary artery bypass graft; AVR = aortic valve replacement; ASD = atrial septal defect; other included ICD lead extraction, ICD replacement/removal.

**Table 2:** Study Outcome Measures. All complications were determined based on electronic health record review for documented diagnosis during ICU stay.

Variable		Group 1 (2017) *n* = 175	Group 2 (2018) *n* = 217	Combined Group 1–2 (2017–2018) *n* = 392	Group 3 (2019) *n* = 158	Comparison Group	95% CI Estimate (Upper, Lower)	*p* Values
HLOS (in days)	Median (IQR)	7.00 (5.00)	6.00 (5.00)	7.00 (6.00)	6.00 (6.00)	2017 to 2019 2018 to 2019 2017–2018 to 2019	1.00 (0.00, 1.00) 0.00 (−5 × 10^−5^, 0.00) 0.00 (0.00, 1.00)	0.071 0.609 0.210
ICU LOS (in days)	Median (IQR)	6.19 (4.88)	5.88 (4.46)	6.01 (4.86)	4.00 (3.00)	2017 to 2019 2018 to 2019 2017–2018 to 2019	2.11 (1.87, 2.93) 1.89 (1.13, 2.12) 2.00 (1.55, 2.28)	<0.001 <0.001 <0.001
30 Day Hospital Readmission Yes	Count (Percentage)	61 (35%)	67 (31%)	128 (33%)	52 (33%)	2017 to 2019 2018 to 2019 2017–2018 to 2019	1.09 (0.67, 1.76) 0.911 (0.57, 1.45) 0.988 (0.67, 1.50)	0.729 0.736 1
Stroke Present	Count (Percentage)	7 (4%)	5 (2%)	12 (3%)	2 (1%)	2017 to 2019 2018 to 2019 2017–2018 to 2019	3.24 (0.60, 32.42) 1.84 (0.30, 19.53) 2.46 (0.54, 22.88)	0.179 0.704 0.369
AKI Present	Count (Percentage)	23 (13%)	27 (12%)	50 (13%)	20 (13%)	2017 to 2019 2018 to 2019 2017–2018 to 2019	1.044 (0.52, 2.10) 0.98 (0.51, 1.92) 1.01 (0.56, 1.86)	1 1 1
Respiratory Failure Present	Count (Percentage)	14 (8%)	18 (8%)	32 (8%)	10 (6%)	2017 to 2019 2018 to 2019 2017–2018 to 2019	1.28 (0.51, 3.34) 1.34 (0.56, 3.34) 1.31 (0.61, 3.08)	0.673 0.553 0.595
Surgical Site Infection Present	Count (Percentage)	4 (2%)	6 (3%)	10 (2.5%)	3 (2%)	2017 to 2019 2018 to 2019 2017–2018 to 2019	1.21 (0.20, 8.38) 1.47 (0.31, 9.21) 1.35 (0.34, 7.74)	1 0.739 0.766
Event (Stroke, AKI, RF, SSI) Present Absent	Count	47 events (7%)	56 events (6%)	104 events (7%)	35 events (5%)	2017 to 2019 2018 to 2019 2017–2018 to 2019	1.23 (0.76, 1.99) 1.18 (0.75, 1.87) 1.21 (0.81, 1.85)	0.424 0.512 0.384

HLOS = hospital length of stay; ICU LOS = intensive care unit length of stay; AKI = acute kidney injury; RF = renal failure; SSI = surgical site infection.

**Table 3:** Amount of vasopressor, blood products and IV fluid administration.

Variable		Group 1 (2017)	Group 2 (2018)	Combined Group 1–2 (2017–2018)	Group 3 (2019)	Comparison Group	95% CI Estimate (Upper, Lower)	*p* Values
Total Norepinephrine (mL)	Median (IQR)	113.46 (239.56)	139.74 (333.01)	120.015 (264.46)	131.25 (302.97)	2017 to 2019 2018 to 2019 2017–2018 to 2019	−24.34 (−71.25, 13.13) −16.8 (−61.92, 29.99) −20.66 (−60.08, 13.19)	0.202 0.431 0.231
Total Phenylephrine (mL)	Median (IQR)	1061.36 (1491.28)	2311.25 (2155.22)	1728.75 (2124.56)	648.75 (834.44)	2017 to 2019 2018 to 2019 2017–2018 to 2019	490.36 (−753.90, 6048.14) 1557.42 (−476.25, 3110.27) 972.90 (−476.25, 2500.90)	0.412 0.214 0.221
Total Milrinone (mL)	Median (IQR)	317.87 (338.30)	310.54 (331.80)	313.17 (333.95)	215.48 (278.62)	2017 to 2019 2018 to 2019 2017–2018 to 2019	103.84 (36.12, 171.30) 88.83 (23.49, 155.49) 95.63 (38.29, 153.48)	<0.01 <0.01 <0.01
Total Epinephrine (mL)	Median (IQR)	165.00 (318.73)	221.32 (330.49)	180.00 (328.11)	180.11 (274.33)	2017 to 2019 2018 to 2019 2017–2018 to 2019	41.39 (−42.25, 108.75) 41.22 (−37.58, 118.00) 41.25 (−22.66, 101.32)	0.271 0.281 0.201
Total Vasopressin (mL)	Median (IQR)	312.00 (909.75)	282.00 (411.00)	300.00 (471.00)	229.50 (342.75)	2017 to 2019 2018 to 2019 2017–2018 to 2019	69.00 (−78.00, 312.00) 72.00 (−48.00 177.00) 71.86 (−39.00, 180.00)	0.344 0.219 0.182
Fresh Frozen Plasma (unit)	Median (IQR)	2.00 (0.75)	1.00 (1.00)	2.00 (1.00)	1.00 (1.00)	2017 to 2019 2018 to 2019 2017–2018 to 2019	0.00 (0.00, 0.00) 0.00 (0.00, 0.00) 0.00 (0.00, 1.00)	0.043 0.831 0.129
Packed Red Blood Cells (unit)	Median (IQR)	4.00 (2.00)	3.00 (2.00)	4.00 (2.00)	2.00(4.00)	2017 to 2019 2018 to 2019 2017–2018 to 2019	0.00 (0.00, 0.00) 0.00 (0.00, 0.00) 0.00 (0.00, 0.00)	0.792 0.665 0.6862
Platelets (unit)	Median (IQR)	2.00 (0.75)	1.00 (0.00)	1.50 (1.00)	1.00 (0.00)	2017 to 2019 2018 to 2019 2017–2018 to 2019	1.00 (0.00, 1.00) NA 0.742 (0.00, 1.00)	0.033 NA 0.095
IV Fluids Intra Op (mL)	Median (IQR)	2481.83 (1256.11)	2167.64 (1042.81)	2290.14 (1153.36)	978.75 (899.63)	2017 to 2019 2018 to 2019 2017–2018 to 2019	1572.65 (1388.23, 1766.17) 1253.40 (1105.80, 1398.33) 1383.05 (1239.96, 1527.90)	<0.001 <0.001 <0.001
IV Fluids ICU (mL)	Median (IQR)	1200.00 (662.50)	1023.75 (800.00)	1100.00 (750.00)	2274.02 (1043.42)	2017 to 2019 2018 to 2019 2017–2018 to 2019	−1117.98 (−1293.80, −949.85) −1224.90 (−1393.80, −1065.88) −1177.88 (−1324.74, −1028.34)	<0.001 <0.001 <0.001
IV Fluids Total (mL)	Median (IQR)	3788.31 (1481.48)	3307.18 (1336.39)	3473.23 (1363.17)	3309.93 (1285.78)	2017 to 2019 2018 to 2019 2017–2018 to 2019	346.65 (83.46, 613.74) 9.96 (−244.67, 255.33) 160.37 (−71.67, 386.17)	0.011 0.951 0.171

## Data Availability

Full protocol and data can be requested from, Penelope Garcia, PEGarcia@Llu.edy, Department of Anestheisology, Loma Linda University Medical Center, Role: research coordinator.
